# Identification of candidate genes for yeast engineering to improve bioethanol production in very high gravity and lignocellulosic biomass industrial fermentations

**DOI:** 10.1186/1754-6834-4-57

**Published:** 2011-12-09

**Authors:** Francisco B Pereira, Pedro MR Guimarães, Daniel G Gomes, Nuno P Mira, Miguel C Teixeira, Isabel Sá-Correia, Lucília Domingues

**Affiliations:** 1 IBB, Institute of Biotechnology and Bioengineering, Centre of Biological Engineering, Universidade do Minho, Campus de Gualtar, 4710-057 Braga, Portugal; 2 IBB, Institute of Biotechnology and Bioengineering, Centre of Biological and Chemical Engineering, Instituto Superior Técnico, Lisbon, Portugal; 3 Department of Bioengineering, Instituto Superior Técnico, Technical University of Lisbon, Avenida Rovisco Pais, 1049-001 Lisbon, Portugal

## Abstract

**Background:**

The optimization of industrial bioethanol production will depend on the rational design and manipulation of industrial strains to improve their robustness against the many stress factors affecting their performance during very high gravity (VHG) or lignocellulosic fermentations. In this study, a set of *Saccharomyces cerevisiae *genes found, through genome-wide screenings, to confer resistance to the simultaneous presence of different relevant stresses were identified as required for maximal fermentation performance under industrial conditions.

**Results:**

Chemogenomics data were used to identify eight genes whose expression confers simultaneous resistance to high concentrations of glucose, acetic acid and ethanol, chemical stresses relevant for VHG fermentations; and eleven genes conferring simultaneous resistance to stresses relevant during lignocellulosic fermentations. These eleven genes were identified based on two different sets: one with five genes granting simultaneous resistance to ethanol, acetic acid and furfural, and the other with six genes providing simultaneous resistance to ethanol, acetic acid and vanillin. The expression of *Bud31 *and *Hpr1 *was found to lead to the increase of both ethanol yield and fermentation rate, while *Pho85*, *Vrp1 *and *Ygl024w *expression is required for maximal ethanol production in VHG fermentations. Five genes, *Erg2*, *Prs3*, *Rav1*, *Rpb4 *and *Vma8*, were found to contribute to the maintenance of cell viability in wheat straw hydrolysate and/or the maximal fermentation rate of this substrate.

**Conclusions:**

The identified genes stand as preferential targets for genetic engineering manipulation in order to generate more robust industrial strains, able to cope with the most significant fermentation stresses and, thus, to increase ethanol production rate and final ethanol titers.

## Background

Fuel ethanol is a renewable and environmentally friendly alternative energy source. Its large scale production has increased significantly over the last few years and is expected to grow even more given the need to reduce the world's dependence of oil [1-3]. Most of the current processes of bioethanol production are based on the use of very high gravity (VHG) fermentations in which highly concentrated media (sugar-cane molasses, starch or grains) are used as substrates [1,3]. The main advantage of VHG technology is the production of very high ethanol titres (usually above 15% v/v), decreasing the cost of the distillation step, which is considered one of the main constraints in the bioethanol industry [3]. In recent years, the interest in the production of bioethanol from alternative residues and, in particular, from agricultural lignocellulosic residues has gained strength. Besides being largely available, these residues do not compete with food resources and are therefore preferable for a sustainable large-scale production of bioethanol [4,5]. To make the lignocellulose present in agricultural residues available, raw materials have to be subjected to a pre-treatment and hydrolysis, during which mostly hemicellulose sugars are released. Under the extreme conditions observed in this pre-treatment step some of these sugars are converted into toxic inhibitors of microbial growth, such as furan derivatives (mostly furfural and 5-hydroxymethylfurfural) and several phenolic compounds (for example, vanillin) [6,7]. Other inhibitory products include acetic acid, which derives from heavily acetylated polymers and is released during pre-treatment and hydrolysis. Acetic acid is frequently the most dominant inhibitor present in plant-biomass hydrolysates [8]. The current knowledge on the mechanisms underlying yeast tolerance to the toxicants present in lignocellulose hydrolysates fermentation, based on molecular studies and genome-wide approaches, was recently reviewed by Liu [9].

The success of lignocellulosic biomass and VHG fermentations is necessarily dependent on the ability of the used yeast strains to cope with the different stresses imposed during these processes. In biomass-based fermentations, yeast cells, besides having to tolerate the presence of the above-referred inhibitors, are also exposed to nutrient starvation and the absence of oxygen [8]. Moreover, the used yeast strains have to remain active under conditions that are near optimal for cellulase activity (pH 5, 40°C to 50°C) and/or secrete cellulase enzymes and co-utilize a variety of sugars at high yields [10]. In VHG fermentations, yeast cells are exposed to a high osmotic pressure in the beginning of the fermentative process, caused by the high sugar concentrations present at that time. Other relevant stresses in VHG fermentations include depletion of some nutrients, a lack of oxygen and an accumulation in the growth medium of high concentrations of ethanol that, together with the elevated levels of other toxic fermentation by-products, become lethal for the fermenting yeast cells [11-14]. The development of yeast strains innately more tolerant to stresses relevant for VHG and/or for biomass fermentations will improve the performance of these processes and contribute to the development of the bioethanol industry.

In this work an integrated approach was undertaken, with the aim of identifying genes required for simultaneous yeast resistance to a high number of fermentation-related stresses. The yeast genes described in large-scale phenotypic analysis as being required for maximal yeast tolerance to ethanol [15], high glucose concentrations (as those found in industrial growth media) [16], acetic acid [17], vanillin [18] and furfural [19] were compared. A set of genes conferring resistance to high concentrations of glucose, acetic acid and ethanol, stresses relevant for VHG fermentations; and to ethanol, acetic acid, furfural and/or vanillin were identified. Comparative fermentative performance analysis under industrially relevant conditions allowed the narrowing down of the number of genes whose expression is required for the maximum performance of VHG fermentations or for the fermentation of wheat straw hydrolysates (WHSs). This therefore revealed suitable candidates for subsequent genetic engineering, with the aim to obtain more robust industrial yeast strains.

## Results

### Identification of *Saccharomyces cerevisiae *genes involved in tolerance to relevant stresses in VHG alcoholic fermentations or in biomass-based fermentations

**Figure 1:**
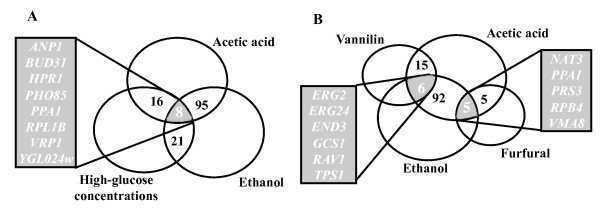
**Comparison of the yeast genes described as determinants of resistance to inhibitory concentrations of (A) ethanol, glucose and acetic acid or of (B) ethanol, acetic acid and furfural or vanillin**. The genes in the intersection of these datasets are highlighted. This comparative analysis was based on published genome-wide phenotypic screenings carried out in the presence of the referred stressors [15-19].

To identify yeast genes that simultaneously confer resistance to inhibitory concentrations of ethanol, glucose and acetic acid or to acetic acid, ethanol, vanillin and/or furfural, we used the results of genome-wide phenotypic screenings carried out in the presence of those stressors [15-19]. Other datasets of determinants of resistance to ethanol and acetic acid were available in the literature [20-23]. However, these studies were not performed using the BY4741 strain, which was the one used to screen the determinants of tolerance to furfural, vanillin and high glucose concentrations [16,18,19], and therefore they were not considered because the genetic background of the yeast strain used is known to have a high impact on the results obtained in large-scale phenotype screenings. Eight genes conferring resistance to ethanol, glucose and acetic acid were identified: *Anp1*, *Bud31*, *Hpr1*, *Pho85*, *Ppa1*, *Vrp1*, *Rpl1B *and *Ygl024w *(Figure 1A). The physiological function of these genes is described in Table 1. No gene providing protection towards acetic acid, ethanol, furfural and vanillin was found. However, six genes common to the dataset of determinants of resistance to acetic acid, vanillin and ethanol (*End3*, *Erg2*, *Erg24*, *Gcs1, Rav1 *and *Tps1*) and five to the dataset of genes required for tolerance to ethanol, acetic acid and furfural (*Nat3, Ppa1*, *Prs3*, *Rpb4 *and *Vma8*) were identified (Figure 1B; Table 1).

**Table 1:** Physiological function of genes shown in Figure 1 as being required for tolerance to inhibitory concentrations of glucose, ethanol and acetic acid or ethanol, acetic acid and vanillin or furfural.

Gene	Function^a^
**Stress: Ethanol, acetic acid and glucose**	

*Anp1*	Subunit of the α-1, 6 mannosyltransferase complex involved in the mannysolation of cell wall proteins
*Bud31*	Protein involved in mRNA splicing
*Hpr1*	Subunit of THO/TREX complexes that couple transcription elongation with mitotic recombination and with mRNA metabolism and export
*Pho85*	Cyclin-dependent kinase involved in the regulation of yeast response to nutrient depletion, environmental stress and cell cycle progression.
*Ppa1*	Proteolipid subunit of the membrane domain of the vacuolar H*-ATPase (V-ATPase)
*Rpl1B*	Component of the large (60S) ribosomal subunit
*Vrp1*	Actin-associated protein involved in cytoskeletal organization and cytokinesis
*Ygl024w*	Unknown Function

**Stress: Ethanol, acetic acid and vanillin**	

*End3*	Protein involved in endocytosis, actin cytoskeletal organization and cell wall morphogenesis
*Erg2*	Sterol isomerase involved in ergosterol biosynthesis
*Erg24*	Sterol reductase involved in ergosterol biosynthesis
*Gcs1*	ADP-ribosylation factor GTPase activating protein, involved in transport from endoplasmic reticulum to Golgi
*Rav1*	Subunit of the RAVE complex which promotes assembly of the V-ATPase
*Tps1*	Synthase subunit of trehalose-6-phosphate synthase/phosphatase complex required for trehalose biosynthesis

**Stress: Ethanol, acetic acid and furfural**	

*Nat3*	Catalytic subunit of the NATB N-terminal acetyltransferase involved in protein acetylation
*Ppa1*	Proteolipid subunit of the membrane domain of the vacuolar H*-ATPase (V*ATPase)
*Rpb4*	RNA polymerase II subunit
*Prs3*	Pyrophosphate synthetase required for nucleotide, histidine and tryptophan biosynthesis
*Vma8*	Peripheral membrane domain subunit of the vacuolar H+-ATPase (V-ATPase)

**^a^**The description of gene function is based on the information available in the *Saccharomyces *Genome Database www.yeastgenome.org. NATB: RAVE: regulator of the ATPase of vacuolar and endosomal membranes.

### Role of genes providing protection against acetic acid, ethanol and glucose stresses in VHG fermentations

**Figure 2:**
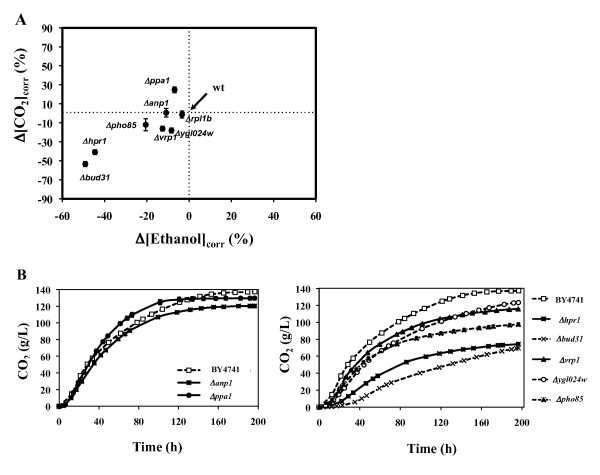
**(A) Comparison between the concentration of CO_2 _(Δ[CO_2_]_corr_) at mid fermentation point (49 h) and of the final amount of ethanol (Δ[Ethanol]_corr_) produced by cells of the parental strain *S. cerevisae *BY4741 and by mutants deleted for the *Anp1*, *Bud31*, *Hpr1*, *Pho85*, *Ppa1*, *Rpl1B, Vrp1 *and *Ygl024w *genes during fermentation of a growth medium optimized for VHG technology**. The Δ[CO_2_]_corr _and Δ[Ethanol]_corr _parameters were calculated using equations 3 and 4, which are detailed in Methods. **(B) **The profile of CO_2 _production by wild-type cells or by the selected deletion mutants (all mentioned above except for Δ*rpl1b *mutant). Those deletion mutants found to start the fermentation at the same time as wild-type cells (shown on left) were separated from those which started the fermentation later (shown on right). Error bars represent the range between independent biological duplicates.

The role in VHG fermentations of the eight genes required for yeast tolerance to inhibitory concentrations of glucose, acetic acid and ethanol (*Anp1*, *Bud31*, *Hpr1*, *Pho85*, *Ppa1*, *Rpl1B, Vrp1 *and *Ygl024w*; Figure 1A) was examined. For this, the fermentation rate and the final concentration of ethanol produced were compared in cells of the parental strain BY4741 and in deletion mutants lacking the above referred genes. The fermentation rate was assessed based on the amount of carbon dioxide (CO_2_) produced at mid-fermentation (49 h). The elimination of the genes under analysis in a non-stressful fermentation carried out in standard yeast extract peptone dextrose (YPD) growth medium (with 2% glucose) did not significantly affect the final ethanol production (no significant differences at 95% confidence level; results not shown). The results obtained in the growth medium optimized for VHG fermentations are summarized in Table 2 and in Figure 2A. Under the oxygen-limiting conditions used in these fermentations, which resemble the typical anaerobic conditions found in large-scale VHG fermentations, the profile of CO_2 _production obtained in wild-type cells and in the different mutants tested was similar to the profile of ethanol formation, which indicates that most of the CO_2 _produced came from the fermentative pathway (results not shown). Taking this into consideration, the profile of CO_2 _production of the wild-type and of the selected deletion mutants is shown in Figure 2B, as it provides a suitable assessment of how the fermentation proceeded in these different strains.

**Table 2:** Effect of the expression of the *Anp1*, *Bud31*, *Hpr1, Pho85*, *Ppa1*, *Rpl1B*, *Vrp1 *and *Ygl024w *genes, required for tolerance to inhibitory concentrations of glucose, acetic acid and ethanol, in VHG fermentation.

Strain	[Ethanol] (g/L)	ΔEthanol (compared to wild-type cells)	[CO_2_] at mid-fermentation (g/L)	ΔCO_2 _(compared to wild-type cells)
BY4741	136 ± 2	0	72 ± 5	0
Δ*anp1*	122 ± 1	-11 ± 1**	67 ± 1	0 ± 4
Δ*bud31*	70 ± 1	-49 ± 1**	14 ± 0	-54 ± 3**
Δ*hpr1*	75 ± 1	-45 ± 1**	28 ± 0	-41 ± 3**
Δ*pho85*	108 ± 0	-21 ± 1**	57 ± 5	-12 ± 6*
Δ*rpl1b*	132 ± 5	-3 ± 3	66 ± 1	-1 ± 4
Δ*vrp1*	117 ± 2	-13 ± 2**	64 ± 1	-16 ± 2**
Δ*vgl024w*	126 ± 1	-8 ± 1**	55 ± 1	-18 ± 3**
Δ*ppa1*	127 ± 1	-7 ± 1**	84 ± 1	25 ± 3**

The comparison of the fermentation profile of wild-type cells and of the deletion mutants was based on the concentration of ethanol produced at the end of the fermentation ([Ethanol] and ΔEthanol) and on the amount of CO_2 _formed ([CO_2_] and ΔCO_2_) at mid-fermentation point (49 h - time taken by the parental strain to reach 50% of the total CO_2 _produced), as described in Methods. The results shown are means of at least two independent experiments and the statistical significance of the results obtained was quantified using a t-test (n = 2). *P <0.05; **P < 0.01.

Parental strain cells produced approximately 136 g/L ethanol (Table 2) from the 304 g/L of glucose that were initially provided in the growth medium, corresponding to an ethanol conversion yield of 87%. Out of the mutants tested only Δ*rpl1b *fermented at a similar rate and produced the same levels of ethanol as wild-type cells (Table 2), leading us to conclude that the *Rpl1B *gene should be dispensable for maximal performance of VHG fermentations. All the other deletion mutants tested produced lower levels of ethanol and/or exhibited reduced fermentation rates (Table 2 and Figure 2A). The highest reduction was observed for Δ*hpr1 *and Δ*bud31 *mutant strains (Table 2, Figure 2A), which produced less than half the amount of ethanol produced by the parental strain. The analysis of the corresponding CO_2 _production profiles showed that fermentation by these mutant cells started significantly later than wild-type cells, which resumed fermentation almost immediately after inoculation (Figure 2B). This observation is consistent with the reported involvement of *Bud31 *and *Hpr1 *in yeast tolerance to inhibitory concentrations of glucose [16]. The fermentation of Δ*hpr1 *and of Δ*bud31 *cells stopped prematurely, leaving almost 130 g/L of glucose in the growth medium (Figure 2B). The analysis of the results obtained for the other deletion mutants indicates that the elimination of *Anp1*, *Pho85*, *Ppa1, Vrp1 *or *Ygl024w *genes does not significantly affect the immediate resumption of fermentation after inoculation. Even so, a significant reduction of the fermentation rate is observed for Δ*pho85*, Δ*vrp1 and *Δ*ygl024w *mutants while an increase is observed for the Δ*ppa1 *mutant. The elimination of *Anp1 *does not affect the fermentation rate but the final ethanol concentration is diminished (Table 2).

### Role of the expression of yeast genes providing resistance to ethanol, acetic acid, furfural or vanillin in the growth and fermentation of a wheat straw hydrolysate

A WSH was prepared following the methodology described by Ruiz *et al. *[24]. Under the used conditions the composition of the solid fraction was 37% glucan, 33% xylan and 27% lignin and the hemicellulosic fraction of the hydrolysate had 1.90 g/L glucose, 15.40 g/L xylose, 2.09 g/L arabinose (both monomeric and oligomeric forms), 1.50 g/L acetic acid, 0.34 g/L formic acid, 0.57 g/L furfural, 0.10 g/L hydroxymethylfurfural (HMF) and a residual concentration of vanillin (below 0.01 g/L). The hydrolysate was supplemented to a final glucose concentration of 50 g/L. The concentrations of the different inhibitors produced in the WSH prepared are consistent with those reported in other studies [4]. From the set of 11 genes providing resistance to ethanol, acetic acid and furfural or vanillin, indicated in Figure 1B, only *Erg2*, *Prs3*, *Rpb4 *and *Vma8 *were required for yeast growth in WSH (Table 3 and Figure S1 in Additional file 1). Consistent with the idea that these genes play no role in yeast growth in the absence of stress, no significant differences in the growth of these deletion mutants and of parental strain cells in YPD growth medium were observed (Figure S1 in Additional file 1). In agreement with the requirement of *Erg2*, *Prs3*, *Rpb4 *and *Vma8 *for growth in the WSH, based on their protective effect against the inhibitors present therein, Δ*erg2*, Δ*prs3*, Δ*rpb4 *and Δ*vma8 *mutants were also unable to grow in a minimal growth medium (MM4) supplemented with the same concentration of inhibitors found in the hydrolysate (Table 3 and Figure S1 in Additional file 1).

**Table 3:** Comparison, by spot assays, of growth of *S. cerevisiae *BY4741 cells and of the 11 deletion mutants that lack the genes found to provide resistance against ethanol, acetic acid and furfural or vanillin.

Strain/medium	WSH^a^	MM4 + inhibitors^b^
**BY4741**	++	++
Δ*prs3*	+	-
Δ*rav1*	++	++
Δ*ppa1*	++	++
Δ*end3*	++	++
Δ*erg24*	++	++
Δ*erg2*	+	-
Δ*nat3*	++	++
*Δvma8*	+	+
*Δgcs1*	++	++
*Δrpb4*	+	+
*Δtps1*	++	++

Cells used to prepare the spots were cultivated in YPD liquid medium until mid-exponential phase (OD_600 nm _= 1.5 ± 0.2) and then applied as spots (4 μL) into the surface of the agar plates containing different growth media. The yeast strains were inoculated in triplicate.^a^Relative to the growth in standard YPD growth medium; ^b ^supplemented with the same mixture of inhibitors found in the hydrolysate, relative to the growth in MM4 medium (without inhibitors). +++ growth; + partial growth; - no growth. MM4: minimal growth medium 4; WSH: wheat straw hydrolysate; YPD: yeast extract peptone dextrose.

**Figure 3:**
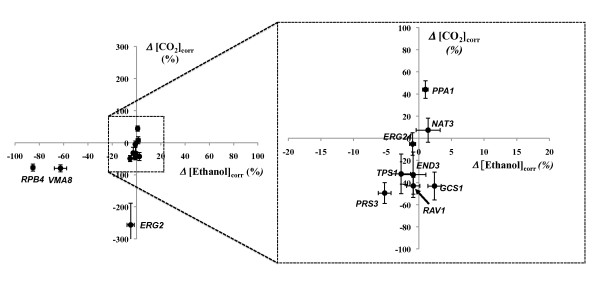
**Comparison between the concentration of CO_2 _(Δ[CO_2_]_corr_) at mid fermentation point (14 h) and of the final concentration of ethanol (Δ[Ethanol]_corr_) produced by cells of the parental strain *S. cerevisae *BY4741 and by the mutants deleted for genes conferring resistance against ethanol, acetic acid and vanillin or furfural in the fermentation of WSH**. The Δ[CO_2_]_corr _and Δ[Ethanol]_corr _parameters were calculated using equations 3 and 4, as described in Methods. Error bars represent the error propagation associated with arithmetic operations used to determine the global relative variation of each mutant strain.

**Figure 4:**
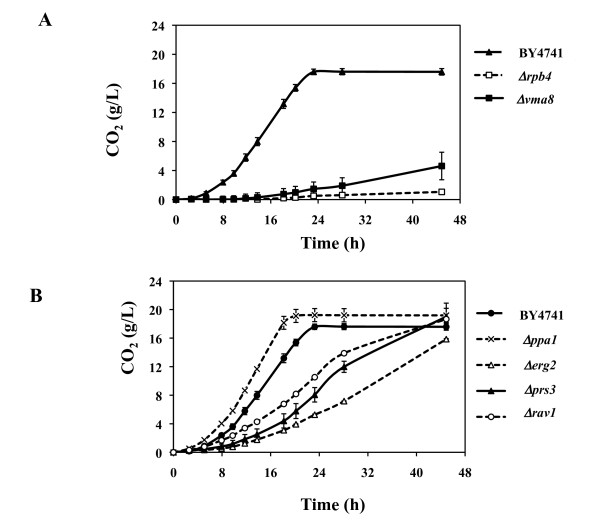
**Profile of CO_2 _production by wild-type cells or by the mutants deleted for genes providing resistance against ethanol, acetic acid and vanillin or furfural**. Those deletion mutants found to produce much lower levels of CO_2 _than those achieved by cells of the parental strain (panel A) were separated from those producing lower, but more similar concentrations (panel B). Error bars represent the range between independent biological duplicates.

The fermentation profile of wild-type *S. cerevisiae *BY4741 cells in the hydrolysate prepared from wheat straw was compared with that of the Δ*end3*, Δ*erg2*, Δ*erg24*, Δ*gcs1*, Δ*nat3*, Δ*ppa1*, Δ*prs3*, Δ*rav1*, Δ*rpb4*, Δ*tps1 *and Δ*vma8 *mutants and the results obtained are summarized in Table 4 and in Figures 3 and 4. The parental strain consumed the 50 g/L of glucose provided in the hydrolysate producing approximately 21 g/L of ethanol (Table 4). Out of the deletion mutants tested, Δ*rpb4 *and Δ*vma8 *were those generating the lowest ethanol concentrations (3 g/L and 7 g/L, respectively), exhibiting also the lowest fermentation rates (Figures 3 and 4A). Consistently, these two mutants were among those whose growth in the hydrolysate was more affected (Table 3 and Figure S1 in Additional file 1). Δ*erg2 *and Δ*prs3 *cells, which were also found to have impaired growth in the hydrolysate (Table 3 and Figure S1 in Additional file 1), did not produce significantly lower ethanol levels compared to wild-type cells (Table 4), but their fermentation rate was found to be much lower (Figures 3 and 4B). This fact may be due to the protective effect that these genes exert against the ethanol that is being accumulated and which is not initially present in the hydrolysate. Despite this, these mutants produced the same levels of ethanol as wild-type cells (Table 4). The Δ*rav1 *mutant, which had not shown impaired growth in the presence of hydrolysate inhibitors (Table 3 and Figure S1 in Additional file 1) and presented the same final ethanol concentration as the wild type strain (Table 4), exhibited a significant decrease in the fermentation rate (Table 4). Interestingly, the fermentation of Δ*ppa1 *cells occurred faster than that carried out by wild-type cells, as had also been observed in the fermentation of the VHG-optimized growth medium (Figures 2B and 4B). However, in contrast to what was observed in the VHG-optimized growth medium, in which a slight reduction in final ethanol production was observed in Δ*ppa1 *cells, these mutant cells produced the same amount of ethanol from the WSH as wild-type cells (Table 4 and Figure 3).

**Table 4:** Effect of the expression of genes required for tolerance to inhibitory concentrations of ethanol, acetic acid and furfural or vanillin in the fermentation of a wheat straw hydrolysate.

Strain	[Ethanol] (g/L)	ΔEthanol (compared to wild-type cells)	[CO_2_] at mid-fermentation (g/L)	ΔCO_2 _(compared to wild-type cells)
BY4741	21 ± 2	0	8 ± 1	0

**Stress: Acetic acid, ethanol and furfural**				

*Δnat3*	22 ± 0	1 ± 2	8 ± 1	7 ± 11
*Δppa1*	21 ± 0	1 ± 0	12 ± 0	44 ± 8 **
*Δprs3*	20 ± 0	-5 ± 1	3 ± 1	-50 ± 9 **
*Δrpb4*	3 ± 0	-85 ± 2 **	0 ± 0	-78 ± 11 **
*Δvma8*	7 ± 1	-63 ± 5 **	0 ± 1	-80 ± 11 **

**Stress: Acetic acid, ethanol and vanillin**				

*Δend3*	21 ± 0	-1 ± 2	5 ± 2	-33 ± 20
*Δerg2*	20 ± 1	-5 ± 3	2 ± 0	-256 ± 68 **
*Δerg24*	21 ± 0	-1 ± 0	8 ± 1	-5 ± 10
*Δgcs1*	22 ± 0	2 ± 1	5 ± 1	-43 ± 13 *
*Δrav1*	21 ± 0	-1 ± 1	4 ± 0	-43 ± 8 **
*Δtps1*	21 ± 0	-3 ± 1	5 ± 1	-32 18

The comparison of the fermentation profile of wild-type cells and of the deletion mutants was based on the concentration of ethanol produced at the end of the fermentation and on the amount of CO_2 _formed at mid-fermentation point (14 h - time taken by the parental strain to reach 50% of the total CO_2 _produced), as described in Methods. The results shown are means of at least two independent experiments and the statistical significance of the results obtained was quantified using a t-test (n = 2). *P < 0.05; **P < 0.01.

## Discussion

In this study, a set of *S. cerevisiae *genes involved in tolerance to relevant stresses in lignocellulosic biomass- or VHG- based fermentations was identified and the requirement of some of these genes for maximal fermentation performance was demonstrated for the first time.

Specifically, the effect of genes involved in tolerance to acetic acid, glucose, ethanol, furfural and vanillin was addressed. The results emerging from chemogenomic screenings have the potential to identify genes that could be interesting targets for subsequent genetic engineering aiming to obtain more robust industrial yeast strains. However, the datasets often reach hundreds of genes, which makes the identification of the best candidates difficult. Furthermore, genes found to be specifically required to confer resistance to individual stresses may not be interesting in a multi-stress environment. In this context, the strategy followed in this study to search for cross-resistance genes provided a straightforward and focused approach. Furthermore, this is a much more realistic approach to the problem since it is clear that it is the combined effect of the different fermentation stressors that poses the greatest challenge to the success of industrial fermentations [8,25-27]. Moreover, as reported by Marks *et al. *[28], studies performed under standard laboratory conditions are inadequate for revealing the mechanisms of metabolic and regulatory changes that occur during industrial fermentation processes. The impact of the combination of relevant stresses that occurs during these fermentations is likely to be more complex, since the yeast cells are subjected to wide variations in diverse environmental factors. It would be interesting to find genes that were able to simultaneously increase yeast tolerance to all the stresses considered in the current study, given the fact that future lignocellulosic hydrolysate formulations will require higher initial sugar concentrations to assure the production of higher bioethanol titers. Since such a gene could not be identified, subgroups of genes were considered separately in the context of lignocellulosic biomass- or VHG- based fermentations.

Out of the eight genes found to contribute simultaneously for yeast tolerance to inhibitory concentrations of glucose, acetic acid and ethanol [15-17], five were demonstrated to be required for maximal fermentation performance in a growth medium optimized for VHG fermentations: *Bud31 *and *Hpr1*, which were found to have a crucial effect in both ethanol yield and fermentation rate; and *Pho85*, *Vrp1 *and *Ygl024w*, which were required for a maximal ethanol production. *Bud31 *and *Hpr1 *encode two proteins involved in general transcription activities; *Bud31 *is required for splicing and associates with yeast spliceossome factors and *Hpr1 *is involved in transcription elongation and mRNA metabolism and export. Significantly, a previous study has successfully engineered the basal yeast transcription machinery to create a strain with increased tolerance to inhibitory concentrations of ethanol and glucose. This strain will, therefore, also have an increased fermentative capacity on sugar-enriched substrates [29]. Interestingly, none of the referred eight genes was among the set of genes whose transcription was reported to be altered in an industrial bioethanol process [30]. Furthermore, the products of these genes were also not found among the proteins whose content was altered during VHG fermentation [31]. Frequently the transcription of a gene that is required for resistance to a given stress is not responsive to that same stress [11,14,32]. This causes gene expression to be a poor predictor of genes important for resistance to fermentation stressors and highlights the usefulness of a chemogenomic analysis that directly addresses the effect of the expression of a given gene in resistance. Moreover, our approach, of identifying key genes common to different relevant stresses in bioethanol fermentations and validating the identified genes under fermentation conditions close to the industrial ones, overcomes some of the constraints of conventional chemogenomic approaches based on laboratory media.

Previous analyses leading to the identification of yeast genes conferring increased resistance to lignocellulose hydrolysis-derived phenolic inhibitors focused mostly on genes related to the specific detoxification of these compounds through their enzymatic conversion into less toxic compounds (reviewed in Liu [9]). Since our focus was the identification of genes whose expression confers simultaneous resistance to these inhibitors, and also to acetic acid and ethanol, it was to be expected that those genes previously found to play very specific roles in yeast resistance to each of these chemical stress inducers would not be present in our dataset. We believe that this integrated approach has the potential to guide the selection of the genes that contribute to the overall viability and fermentative capacity of yeast cells under multiple stress conditions. Among the genes providing resistance to ethanol, acetic acid and vanillin or furfural, *Erg2*, *Prs3*, *Rpb4 *and *Vma8 *were found, for the first time, to contribute to maximal yeast cell growth and fermentation rate in WSH, while *Rav1 *contributed only to reaching maximal fermentation rate. *Rav1 *and *Vma8 *are both involved in the assembly and function of the vacuolar membrane H^+^-ATPase (V-ATPase): Rav1 is a subunit of the RAVE complex (Regulator of the ATPase of Vacuolar and Endosomal membranes) which promotes assembly of V-ATPase holoenzyme [33], and *Vma8 *encodes the subunit D of the V1 peripheral membrane domain of the enzyme. V-ATPase plays a crucial role in the maintenance of internal pH within physiological values, especially under conditions of stress that induce intracellular acidification, as is the case of acetic acid and ethanol stress [15,34]. Consequently, V-ATPase was identified as a crucial determinant of resistance to these two chemicals [15,17]. *Erg2 *encodes one of the key enzymes involved in ergosterol biosynthesis. A decrease in the transcript levels of ergosterol biosynthetic genes was reported previously to occur during bioethanol [30] and winemaking [35] processes, possibly as a response to the lack of oxygen. The activation of ergosterol biosynthesis seems to be one of the reasons why the frequent addition of oxygen is used to increase yeast viability and fermentation quality in the winemaking processes. Several studies have correlated ergosterol (the major sterol in the plasma membrane of *S. cerevisiae*) with yeast tolerance to stress, particularly against ethanol [36,37], indicating a prominent role of this lipid in stabilizing membrane lipids and proteins against the detrimental effects of ethanol. Remarkably, it was recently demonstrated that V-ATPase activity is reduced in mutants devoid of *Erg2 *expression [38]. The *Erg24 *gene, encoding a C-14 sterol reductase also involved in ergosterol biosynthesis, was also identified as a common determinant of resistance to ethanol, acetic acid, vanillin and furfural; however, the expression of this gene was dispensable for yeast growth in the WSH and only had a slight effect on the fermentation performance of this substrate. This may be due to the fact that the deletion of *Erg2 *and *Erg24 *lead to the accumulation of different sterols in the yeast plasma membrane.

## Conclusions

With this study, we successfully narrowed down the number of genes previously identified through genome-wide screenings whose genetic manipulation is promising in the context of bioethanol process optimization. The focused and more realistic approach exploited in this study allowed us to confirm the practical importance of a set of genes for maximal fermentation performance in a growth medium optimized for VHG and/or lignocellulosic biomass industrial fermentations. These results expand our understanding of the genes and underlying molecular mechanisms that are directly involved in yeast tolerance and response to the multiple stresses occurring during bioethanol fermentations under industrially relevant conditions. The use of genetic engineering approaches to increase the expression of the selected genes in industrial strains is the next logical step, to find out whether these manipulations may lead to the generation of more robust industrial yeast strains, able to cope with the most significant fermentation stresses and, thus, to increase ethanol production rate and final ethanol titers.

## Methods

### Strains and growth media

The parental strain *S. cerevisiae *BY4741 *(MATa, his3*Δ*1*, *leu2*Δ*0*, *met15*Δ*0 *and *ura3*Δ*0*) and the 18 derived deletion mutant strains used in this study *(*Δ*anp1*, Δ*bud31*, Δ*end3*, Δ*erg2*, Δ*erg24*, Δ*gcs1*, Δ*hpr1*, Δ*nat3*, Δ*pho85*, Δ*ppa1*, Δ*prs3*, Δ*vrp1*, Δ*rav1*, Δ*rpb4*, Δ*rpl1b*, Δ*tps1*, Δ*vma8 *and Δ*ygl024w*) were acquired from the EUROSCARF collection.

VHG fermentations were carried out in a growth medium that contains, per liter: 304 g glucose, 44.3 g corn steep liquor (CSL), 2.3 g urea, 3.8 g magnesium sulfate heptahydrate (MgSO_4_·7H_2_O) and 0.03 g copper sulfate pentahydrate (CuSO_4_·5H_2_O). The pH of the medium was adjusted to 5.5 using 1 M sodium hydroxide. CSL was kindly provided by a starch manufacturer (COPAM, Lisbon, Portugal) and its manipulation in the laboratory as well as its detailed composition have been previously described by Pereira *et al. *[39]. Control fermentations were performed in YPD growth medium that contains 2% (w/v) glucose, 2% (w/v) bactopeptone and 1% (w/v) yeast extract. The MM4 growth medium used to test the susceptibility of yeast cells to the inhibitors found in WHSs contained, per liter, 20 g glucose, 2.65 g ammonium sulfate, 1.7 g yeast nitrogen base without amino acids and ammonium sulfate, 20 mg methionine, 20 mg histidine, 60 mg leucine and 20 mg uracil. Solid YPD and MM4 growth media were obtained by supplementing the liquid medium with 2% (w/v) agar.

### Preparation of the WSH

A lignocellulosic WSH was prepared following the method described by Ruiz *et al. *[24]. Briefly, the milled wheat straw (with particle size distribution of: > 1 mm, 10%; between 1 mm and 0.5 mm, 40%; between 0.5 mm and 0.3 mm, 40%; < 0.3 mm, 10%) and water were mixed in order to obtain a ratio 10:1 liquid/solid and treated for 30 min in a 3.75 L stainless steel reactor, at 180°C. After hydrolysis, the liquid phase (hemicellulosic liquor) was collected by filtration and stored at -20°C. Prior to its use for fermentation, the hemicellulosic liquor was centrifuged for 10 min at 4800 *g *(4°C) to remove the solid fraction and then sterilized by filtration. The liquid phase of the hemicellulosic liquor was supplemented with glucose (up to a final concentration of 50 g/L) to improve the ethanol yields and with 240 mg/L leucine, 80 mg/L histidine, 80 mg/L methionine and 80 mg/L uracil to account for the auxotrophies of the BY4741 strain. The pH of this liquid fraction was finally adjusted to 5.5 with 10 M sodium hydroxide. The concentrations of glucose, xylose, arabinose, acetic acid, formic acid, furfural, vanillin and HMF in the WSH prepared, as described above, were quantified by high performance liquid chromatography (HPLC). Glucose, xylose, acetic acid and formic acid were quantified upon separation of an aliquot of the hydrolysate in a Varian MetaCarb 87H column, eluted at 60°C with 0.005 M sulfuric acid, at a flow rate of 0.7 mL/min. The peaks corresponding to glucose, xylose and arabinose were detected using a refractive index detector, whereas acetic acid and formic acid were detected using an UV detector set at 210 nm. Furfural, vanillin and HMF were quantified upon separation of an aliquot of the hydrolysate in a Macherey-nagel C18 column, eluted with 20% acetonitrile to 80% water at a flow rate of 0.9 mL/min. Peak detection was performed using an UV detector set at 276 nm.

### Fermentations in VHG-optimized medium or in WSH

Cells used to inoculate the optimized VHG-growth medium or the WSH were cultivated at 30°C for 24 h, with orbital agitation (150 rpm), in YPD growth medium (supplemented with 240 mg/L leucine, 80 mg/L histidine, 80 mg/L methionine and 80 mg/L uracil). After that, cells were harvested by centrifugation (10 min, 4800 *g*, 4°C) and the pellet was resuspended in ice-cold 0.9% (w/v) sodium chloride to obtain 200 mg/mL fresh yeast. This concentrated cell suspension was used to inoculate 35 mL of the VHG-optimized growth medium or the WSH, with a cellular concentration of 1 × 10^8 ^cells/mL. Fermentations were carried out in 100 mL Erlenmeyer flasks fitted with perforated rubber stoppers enclosing glycerol-filled air-locks (to permit CO_2 _exhaustion while avoiding the entrance of air). Prior to inoculation, both media were aerated by stirring with a magnetic bar (length 3 cm) at > 850 rpm for 20 min. Under these conditions the oxygen concentration in the growth media was higher than 95% of air saturation. The fermentation was followed by measuring the reduction of mass loss resulting from CO_2 _production. At each time point, the standard deviation between replicates was less than 2% of the average value for the CO_2 _production. The concentrations of glucose, glycerol and ethanol in the growth media throughout the fermentations were quantified by HPLC. For this, an aliquot of the culture supernatant was separated on a Varian MetaCarb 87H column and eluted at 60°C with 0.005 M sulfuric acid at a flow rate of 0.7 mL/min. Peak detection was performed using a refractive-index detector.

### Comparative analysis of the fermentation profile of VHG-optimized growth medium or WSH by wild-type *S. cerevisae *BY4741 cells and selected deletion mutants

The fermentation profile in VHG-optimized medium or in the WSH of the parental strain BY4741 was compared with that of selected deletion mutants based on two kinetic parameters; the concentration of ethanol present at the end of the fermentation and the amount of CO_2 _produced at mid-fermentation point, that is, the time at which the amount of CO_2 _produced by wild-type cells achieved half of the total produced. For each deletion mutant these two fermentation parameters were compared with those of the parental strain using equations 1 and 2:

(1) $$\Delta Ethanol_{VHG\;or\;WSH}=\frac{{\left[Ethanol\right]}_{mutantstrain}-{\left[Ethanol\right]}_{wild-type}}{{\left[Ethanol\right]}_{wild-type}}$$

(2) $$\Delta CO_{2\;\;VHG\;or\;WSH}=\frac{{\left[CO_{2}\right]}_{mutantstrain}-{\left[CO_{2}\right]}_{wild-type}}{{\left[CO_{2}\right]}_{wild-type}}$$

To take into account that the elimination of a gene may affect the fermentation performance even in the absence of stress, the fermentation profile of the parental strain BY4741 and of the 18 deletion mutants used in this study was compared in standard YPD growth and the values of Δ[Ethanol]_VHG or WSH _and Δ[CO_2_]_VHG or WSH _were corrected using equations 3 and 4:

(3) $$\Delta {\left[Ethanol\right]}_{corr}=\frac{{\left[\Delta \;Ethanol\right]}_{VHG\;or\;WSH}}{1-\Delta {\left[Ethanol\right]}_{YPD}}$$

(4) $$\Delta {\left[CO2\right]}_{corr}=\frac{\Delta {\left[CO_{2}\right]}_{VHG\;or\;WSH}}{1-\Delta {\left[CO_{2}\right]}_{YPD}}$$

A t-test (one-way ANOVA) was used to assess the statistical significance of the results.

### Comparative analysis of the growth of wild-type cells and of the selected deletion mutants in WSH using spot assays

Growth of wild-type BY4741 cells in the WSH was compared with that of the deletion mutants Δ*end3*, Δ*erg2*, Δ*erg24*, Δ*gcs1*, Δ*nat3*, Δ*ppa1*, Δ*prs3*, Δ*rpb4*, Δ*vma8*, Δ*rav1 *and Δ*tps1 *using spot assays. For this, cells were cultivated at 30°C with orbital agitation (150 rpm) in YPD liquid medium until mid-exponential phase (OD_600 _of 1.5 ± 0.2) and then diluted to obtain a cell suspension with a standardized OD_600 nm _of 0.1 ± 0.02. Four microliters of this cellular suspension were applied as spots onto the surface of plates containing the WSH supplemented with 2% agar. The susceptibility of wild-type cells and of the deletion mutants was also compared in MM4 growth medium supplemented or not with the same mixture of inhibitors found in the WSH, that is 1.5 g/L acetic acid, 0.34 g/L formic acid, 0.57 g/L furfural and 0.1 g/L HMF. The pH of the MM4 growth medium was adjusted to pH 5.5 with sodium hydroxide prior to autoclaving. The cell suspensions used to inoculate the MM4 growth media plates were obtained as described above.

## Competing interests

The authors declare that they have no competing interests.

## Authors' contributions

FP participated in the design of experiments, collected the data and drafted the manuscript. DGG participated in data collection. PMR, NPM and MCT participated in the design of experiments and helped write the manuscript. ISC and LD coordinated the research and helped to finalize the manuscript. All authors read and approved the final manuscript.

## Supplementary Material

Additional File 1 **Figure S1**. **Comparison, by spot assays, of the growth of *S. cerevisiae *BY4741 cells and of the 11 deletion mutants that lack the genes found to provide resistance against ethanol, acetic acid and furfural or vanillin. (A) **in wheat straw hydrolysate; **(B) **in standard YPD growth medium and **(C, D) **in MM4 medium supplemented, or not, with the same mixture of inhibitors found in the hydrolysate. Cells used to prepare the spots were cultivated in YPD liquid medium until mid-exponential phase (OD_600 nm _= 1.5 ± 0.2) and then applied as spots (4 μL) into the surface of the agar plates containing different growth media. The yeast strains were inoculated in triplicate and always in the same order: **1**. BY4741; **2**. Δ*prs3*; **3**. Δ*rav1*; **4**. Δ*ppa1*; **5**. Δ*end3*; **6**. Δ*erg24*; **7**. Δ*erg2*; **8**. Δ*nat3*; **9**. Δ*vma8*; **10**. Δ*gcs1*; **11**. Δ*rpb4*; **12**. Δ*tps1*. Click here for file
