# Supplementary figures and images for: Identification of candidate genes for yeast engineering to improve bioethanol production in very high gravity and lignocellulosic biomass industrial fermentations
Source: Biotechnol Biofuels. 2011 Dec 9;4:57. doi: 10.1186/1754-6834-4-57 (PMC3287136; doi:10.1186/1754-6834-4-57)

**A) Wheat straw hydrolysate**

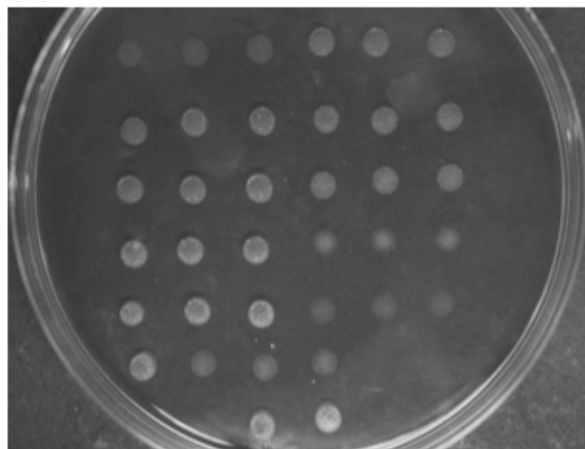

**B) YPD**

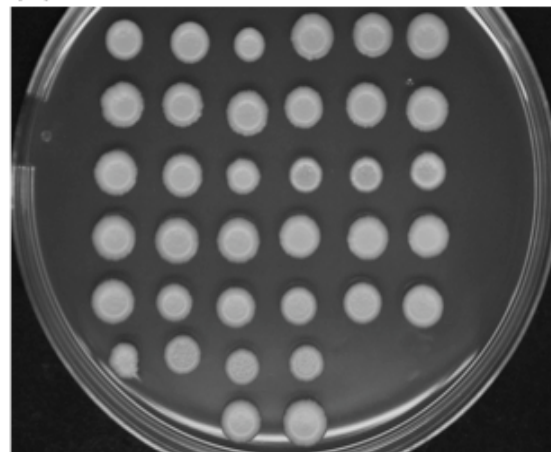

**C) MM4**

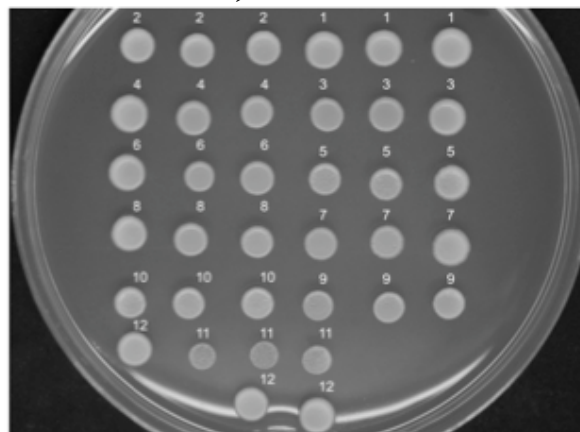

**D) MM4 + inhibitors**

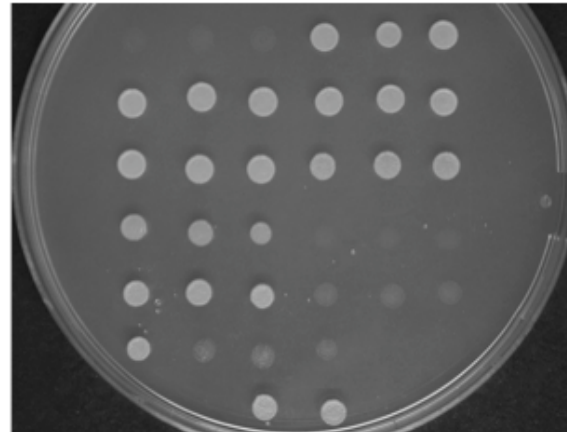

**Figure S1**

Supplement: Additional File 1 — Figure S1. Comparison, by spot assays, of the growth of S. cerevisiae BY4741 cells and of the 11 deletion mutants that lack the genes found to provide resistance against ethanol, acetic acid and furfural or vanillin. (A) in wheat straw hydrolysate; (B) in standard YPD growth medium and (C, D) in MM4 medium supplemented, or not, with the same mixture of inhibitors found in the hydrolysate. Cells used to prepare the spots were cultivated in YPD liquid medium until mid-exponential phase (OD600 nm = 1.5 ± 0.2) and then applied as spots (4 μL) into the surface of the agar plates containing different growth media. The yeast strains were inoculated in triplicate and always in the same order: 1. BY4741; 2. Δprs3; 3. Δrav1; 4. Δppa1; 5. Δend3; 6. Δerg24; 7. Δerg2; 8. Δnat3; 9. Δvma8; 10. Δgcs1; 11. Δrpb4; 12. Δtps1. [file 1754-6834-4-57-S1.PDF]
